# Translingual neural stimulation induced changes in intra- and inter-network functional connectivity in mild-moderate traumatic brain injury patients

**DOI:** 10.3389/fnhum.2025.1481474

**Published:** 2025-01-24

**Authors:** Daniel Y. Chu, Jiancheng Hou, Thomas Hosseini, Veena A. Nair, Nagesh Adluru, Yuri Danilov, Kurt A. Kaczmarek, Mary E. Meyerand, Mitchell Tyler, Vivek Prabhakaran

**Affiliations:** ^1^Department of Radiology, School of Medicine and Public Health, University of Wisconsin-Madison, Madison, WI, United States; ^2^Research Center for Cross-Straits Cultural Development, Fujian Normal University, Fuzhou, Fujian, China; ^3^Department of Kinesiology, University of Wisconsin-Madison, Madison, WI, United States; ^4^Department of Biomedical Engineering, University of Wisconsin-Madison, Madison, WI, United States

**Keywords:** traumatic brain injuries, translingual neural stimulation, network functional connectivity, Sensory Organization Test, Dynamic Gait Index

## Abstract

**Introduction:**

Mild-to-moderate traumatic brain injury (mmTBI) that lead to deficits in balance and gait are difficult to resolve through standard therapy protocols, and these deficits can severely impact a patient's quality of life. Recently, translingual neural stimulation (TLNS) has emerged as a potential therapy for mmTBI-related balance and gait deficits by inducing neuroplastic changes in the brain gray matter structure. However, it is still unclear how interactions within and between functional networks in brain are affected by TLNS. The current study aimed to extend our previous resting-state functional connectivity (RSFC) study investigating the effects of TLNS intervention on outcome measures related to gait and balance.

**Methods:**

An experimental PoNS device was utilized to deliver the TLNS. The 2-week TLNS intervention program, specifically stimulation during focused physical therapy focused on recovery of gait and balance, included twice-daily treatment in the laboratory and the same program at home during the intervening weekend. The resting-state fMRI datasets at pre- and post-interventions were collected by 3T MRI scanner with nine mmTBI patients. All participants also received both Sensory Organization Test (SOT) and Dynamic Gait Index (DGI) testing pre- and post-intervention as part of the behavioral assessment.

**Results:**

Compared to baseline, TLNS intervention led to statistically significant improvements in both the SOT [*t*_(8)_ = 2.742, *p* = 0.028] and the DGI [*t*_(8)_ = 2.855, *p* = 0.024] scores. Moreover, significant increases in intra- and inter-network RSFC were observed, particularly within the visual, default mode, dorsal attention, frontoparietal (FPN), and somatosensory (SMN) networks. Additionally, there were significant correlations between the SOT and inter-network FC [between FPN and SMN, *r*_(9)_ = –0.784, *p* = 0.012] and between the DGI and intra-network FC [within SMN, *r*_(9)_ = 0.728, *p* = 0.026].

**Discussion:**

These findings suggest that TLNS intervention is an effective in increasing somatosensory processing, vestibular-visual interaction, executive control and flexible shifting, and TLNS may be an effective approach to inducing brain network plasticity and may serve as a potential therapy for mmTBI-related gait and balance deficits.

## 1 Introduction

Traumatic brain injury (TBI) is a form of externally acquired injury to the brain that can produce cognitive, emotional, social, and physical deficits (McDonald, 2013). One of the most common complications is headache, which has been shown to contribute to anxiety and depression in patients (Mofatteh, 2021). Additionally, a general deficit that can be seen in patients suffering from mild-to-moderate TBI (mmTBI) is balance injury (Li Y. et al., 2013). Physical therapy, a vestibular-based therapeutic exercise focusing on gait and balance training, is the typical standard of care for treating these functional deficits and promoting neurological health in mmTBI (Gottshall, 2011). However, this vestibular rehabilitation therapy also has a limited effect on its persistent functional recovery (Han et al., 2011).

Translingual neural stimulation (TLNS) is a novel therapeutic intervention that combines superficial electrical stimulation of the facial and trigeminal nerves with physical therapy focused on reducing balance and gait deficits (Danilov et al., 2015). TLNS is provided via the Portable Neuromodulation Stimulator (PoNS^®^, Helius Medical Technologies, Newtown, Pennsylvania, USA), a compact electrical apparatus that delivers comfortable electrical stimulation to the surface of the tongue (Tyler et al., 2019). The stimuli induce action potentials in the facial and trigeminal nerves that subsequently propagate to the cerebellum and brainstem, which may ultimately lead to functional changes in brain structures (Wildenberg et al., 2011).

A previous study showed that TLNS combined with physical therapy can affect the rehabilitation outcomes (Bolognini et al., 2009; Motamed Vaziri et al., 2014), and non-invasive brain stimulation can affect neural excitability and facilitate motor skill learning (Li et al., 2015). A study by our group showed that TLNS combined with PT can significantly improve outcomes in patients with degenerative neurologic disease, spinal cord injury or stroke (Tyler et al., 2014).

Studies on TLNS treatment with mmTBI patients have shown that targeted physical rehabilitation combined with neurostimulation can reduce symptoms and benefit neuronal recovery (e.g., the cerebellum, brainstem, and pons) (Danilov et al., 2015; Wildenberg et al., 2011). A previous voxel-based morphometry study found that the gray matter volume (GMV) significantly increased in the temporal and cerebellar regions, which are responsible for the automatic processing of visual motion, motor control, balance, and gait. However, significant decreases in the frontal and occipital regions, involved with effortful processing of vision, motor plan or control, were also seen. Our recent resting-state functional magnetic resonance imaging (fMRI) study further revealed increased resting state functional connectivity (RSFC) in specific regions-of-interest (ROIs), namely the left postcentral gyrus, left inferior parietal lobule, and between the right culmen and right declive (Hou et al., 2022), indicating positive effects of TLNS treatment on brain plasticity of somatosensory and visual inputs, visual-vestibular interactions, and balance control in mmTBI patients.

However, seed-based RSFC only provides information regarding functional interactions between specific brain regions; its analysis usually requires a priori region of interest (ROI) definition, and the results strongly depend upon and are limited by the ROI chosen (Smith et al., 2011; Tian et al., 2012). Moreover, the human brain is organized as a network, where the local architecture (e.g., short-range connections) is integrated with the large architecture (e.g., long-range connections) in order to support high-level cognitive function (Li et al., 2018; Park and Friston, 2013). Thus, network-based approaches offer a broader perspective on brain activity by capturing the interactions across the entire brain, without the need to restrict the analysis to predefined regions. Some clinical practices have employed the resting-state functional network approach to stroke (Falcon et al., 2016), schizophrenia (Bassett et al., 2012) and intracranial space-occupying lesions (Guan et al., 2022).

The present study specifically examines the intra- and inter-network functional connectivity changes from before (pre-) and after (post-) TLNS intervention in mmTBI patients. The subsequent functional network changes were correlated to changes in behavioral testing of gait and balance before and after TLNS intervention. We hypothesized that TLNS treatment in mmTBI patients would lead to increased RSFC measures between motor and sensory networks with a corresponding observable increase in balance and gait.

## 2 Methods

### 2.1 Participants

This study was performed between June and October, 2016, and the participants were recruited through print and radio advertising. The behavioral testers and participants were blinded to the participants' intervention status. Nine participants with mmTBI were involved (3 males, 6 females; 43–62 years old, mean age = 53.11 ± 6.60 years (see Table 1). Their mmTBI occurred at least 1 year before enrollment. Participants had previously participated in physical therapy, had reached a plateau in their functional recovery, and still scored at least 16-points below normal (after age adjustment) on the Sensory Organization Test (SOT), a quantitative dynamic posturographic analysis system (NeuroCom^®^). Their mmTBI diagnoses were made according to the guidelines established by the Veterans Affairs/Department of Defense (Management of Concussion/mTBI Working Group, 2009). This study was approved by the Institutional Review Board at School of Medicine and Public Health, University of Wisconsin–Madison. All participants in this study provided informed consent before the experiments.

**Table 1:** The characteristics of mmTBI patients.

**Subjects**	**Gender**	**Age**	**Education (years)**	**SOT**		**DGI**	
				**Baseline**	**Post-intervention**	**Baseline**	**Post-intervention**
1	Male	50	20	57.00	48.00	22	24
2	Female	44	16	10.00	33.00	10	12
3	Female	53	13	60.00	84.00	24	24
4	Female	53	17	35.00	63.00	17	19
5	Male	62	20	43.00	57.00	19	20
6	Female	55	20	40.00	65.00	17	22
7	Female	43	14	49.00	40.00	22	23
8	Female	47	13	38.00	66.00	23	23
9	Male	60	12	55.00	79.00	21	21

SOT, Sensory Organization Test; DGI, Dynamic Gait Index.

All nine participants received HFP or LFP stimulation. Inclusion criteria were: Participants were able to walk independently for at least 20 min, had access to a treadmill while not in the clinic, and had no medication changes for at least 3 months before the study. Exclusion criteria were: No additional medical problems such as oral health problems, unmanaged hypertension, diabetes, chronic infectious disease, a penetrating head injury, craniotomy, or refractory subdural hematoma, history of treatment for cancer other than basal cell carcinoma within the past year, neurological disorders other than those attributed to their primary diagnosis, non-removable metal orthodontic devices, or oral cavity piercings that could interfere with TLNS use. Additionally, long-term use of psychoactive or psychostimulant medications could compromise participants' ability to comprehend and perform study activities was also grounds for exclusion, as was the presence of a pacemaker or elevated risk for cardiovascular events. Furthermore, participants with a history of substance use disorder were not included. In the end, individuals with a lower extremity biomechanical prosthetic, history of seizures, or a “severe” score in any of the attention, memory, or executive functions categories on the Cognitive Linguistic Quick Test (CLQT) were also excluded (Tyler et al., 2019).

### 2.2 Intervention

An experimental PoNS device (version 2.5) was utilized to deliver the TLNS, using 143 electrodes on the tongue array to administer 19-volt amplitude-controlled, pulse-width modulated, unbalanced biphasic pulses to the anterior and superior surface of the tongue. The waveform delivers a zero net direct current to minimize the potential for tissue irritation (Tyler et al., 2019). This experimental PoNS device produced the same electrical stimulation as a commercially available PoNS device (Helius Medical Technologies), which received regulatory clearance for treating balance and gait disorders arising from mmTBI and multiple sclerosis (MS) in Canada, MS in the USA, and all neurologically-based balance and gait disorders in Australia. The 2-week TLNS intervention program, specifically stimulation during focused physical therapy focused on recovery of gait and balance, included twice-daily treatment in the laboratory and the same program at home during the intervening weekend. The participants also received physical exercise training focusing on motor coordination and mobility as part of the TLNS training. All participants worked with a physical therapist twice daily for a total of 1-h (each session being 30 min in length) to perform the different training modules (two balance, two gait, one warm-up, one movement control exercise, one breathing and awareness training [BAT]), followed by a BAT session completed independently at home (Ptito et al., 2021).

### 2.3 Behavioral testing

All participants received both SOT and the Dynamic Gait Index (DGI) testing at baseline (before, or pre-intervention), and after 2 weeks of twice-daily intervention (post-intervention) as part of the behavioral assessment battery. The SOT is an objective and automated testing of sensory-motor integration that assesses the levels of somatosensory, visual, and vestibular balance. The DGI is a clinician-scored examination of eight facets of gait and is scored from 0 (worst) to 24 (normal). A score change of 3 points is generally considered clinically significant (Tyler et al., 2019).

### 2.4 MRI acquisition

Both resting-state fMRI and T1 structural MRI data (3T MRI GE750 scanner, GE Healthcare, Waukesha, Wisconsin, USA) were acquired at baseline (pre-) and immediately after a 2-week (post-) TLNS intervention. Ten minutes of eyes-closed resting-state fMRI was acquired with the following parameters: repetition time (TR) = 2,000 ms, echo time (TE) = 22 ms, flip angle (θ) = 60°, field of view (FOV) = 100 mm and matrix size = 100 × 100, voxel size = 3.5 mm^3^ isotropic. The anatomical data scan was acquired using a T1-weighted, three-dimensional, gradient-echo pulse-sequence (MPRAGE) with TR = 8,132 ms, TE = 3.18 ms, TI = 450 ms, flip angle θ = 12°, FOV = 100 mm and matrix size = 100 × 100, and in-plane resolution = 1 mm^2^ isotropic. Participants' head motion was minimized by MRI compatible foam pads.

### 2.5 Data preprocessing

Preprocessing of RS-fMRI data was performed using the Data Processing and Analysis of Brain Imaging (DPABI) toolbox version 6.0 (http://rfmri.org/dpabi), which includes the sub-toolbox of Data Processing Assistant for Resting-state fMRI Advanced Edition toolbox (DPARSF V5.3) (Chang and Glover, 2010; Yan et al., 2016). DPARSF is an easy plug-in software tool that works with Statistical Parametric Mapping (SPM, version 12) (https://www.fil.ion.ucl.ac.uk/spm/software/spm12/) integrated in Matlab (Chao-Gan and Yu-Feng, 2010). The first five volumes were discarded to allow the magnetization to approach a dynamic equilibrium so the participants could become accustomed to the scanner noise. The preprocessing steps, in order, included slice timing correction, realignment, regressing out head motion parameters (scrubbing with Friston 24-parameter model regression; bad time points were identified using a threshold of frame-wise displacement >0.2 mm, and 1 volume before and 2 volumes after at the individual-subject level as well as accounting for head motion at the group-level (i.e., covariate analysis) (Power et al., 2012; Yan et al., 2013, 2016), normalization (spatial normalization to the MNI template, resampling voxel size of 3.5 × 3.5 × 3.5 mm^3^), and smoothing (a spatial Gaussian filter of 4 mm full-width at half maximum was used) (Chao-Gan and Yu-Feng, 2010; Kuhn et al., 2012). The temporal correlations as spontaneous neural connectivity were calculated to quantify RSFC. Additionally, the symmetric correlation matrices for a 160 × 160 network with the Dosenbach atlas, which defines 160 regions or nodes distributed across the brain (Dosenbach et al., 2010), were generated for each participant for pre- and post-interventions. Based on the matrix, each participant had a total of 25,600 unique pairwise functional connections at each pre- and post-intervention stage. Only half of the pairwise functional connections within the network were used for further brain network construction because the top right half and bottom left half were the same in the matrix.

### 2.6 Network functional connectivity construction

Network functional connectivity (FC) was constructed for each participant with the DPABINet V1.1 that was integrated in DPABI V6.0. The 160 nodes in the Dosenbach atlas were classified into eight subnetworks using the Yeo et al. classification: visual network (VN), somatosensory network (SMN), ventral attention network (VAN), dorsal attention network (DAN), default mode network (DMN), subcortical network (SC), frontoparietal network (FPN), and cerebellum network (CN) (Yeo et al., 2011). Based on the 160 × 160 matrix produced at the preprocessing step, the network FC for any pair of nodes was calculated as a Pearson's linear correlation coefficient, which was then Fisher-*z* transformed to derive a symmetric z score matrix that represented the functional connectivity network.

### 2.7 Statistical analysis

A paired *t*-test between pre- vs. post-intervention was performed to compare the changes exhibited in the network FC, and two-tailed permutation testing in DPABINet was used with 5,000 permutations (Winkler et al., 2016). The head motion (mean framewise displacement [FD]) was included in the model as a covariate. False discovery rate (FDR) corrected *p* < 0.05 was used for multiple comparisons correction, and the results were visualized using DPABINet Viewer. The correlation analysis between SOT (or DGI; post- minus pre-) and RSFC (post- minus pre-) was corrected at *p* < 0.05 with IBM SPSS version 23.

## 3 Results

### 3.1 Behavioral scores

TLNS intervention led to significant increases in both SOT scores (*t*_(8)_ = 2.742, *p* = 0.028) and DGI scores (*t*_(8)_ = 2.855, *p* = 0.024) from pre-intervention to post-intervention.

### 3.2 Intra-network functional connectivity

**Figure 1:**
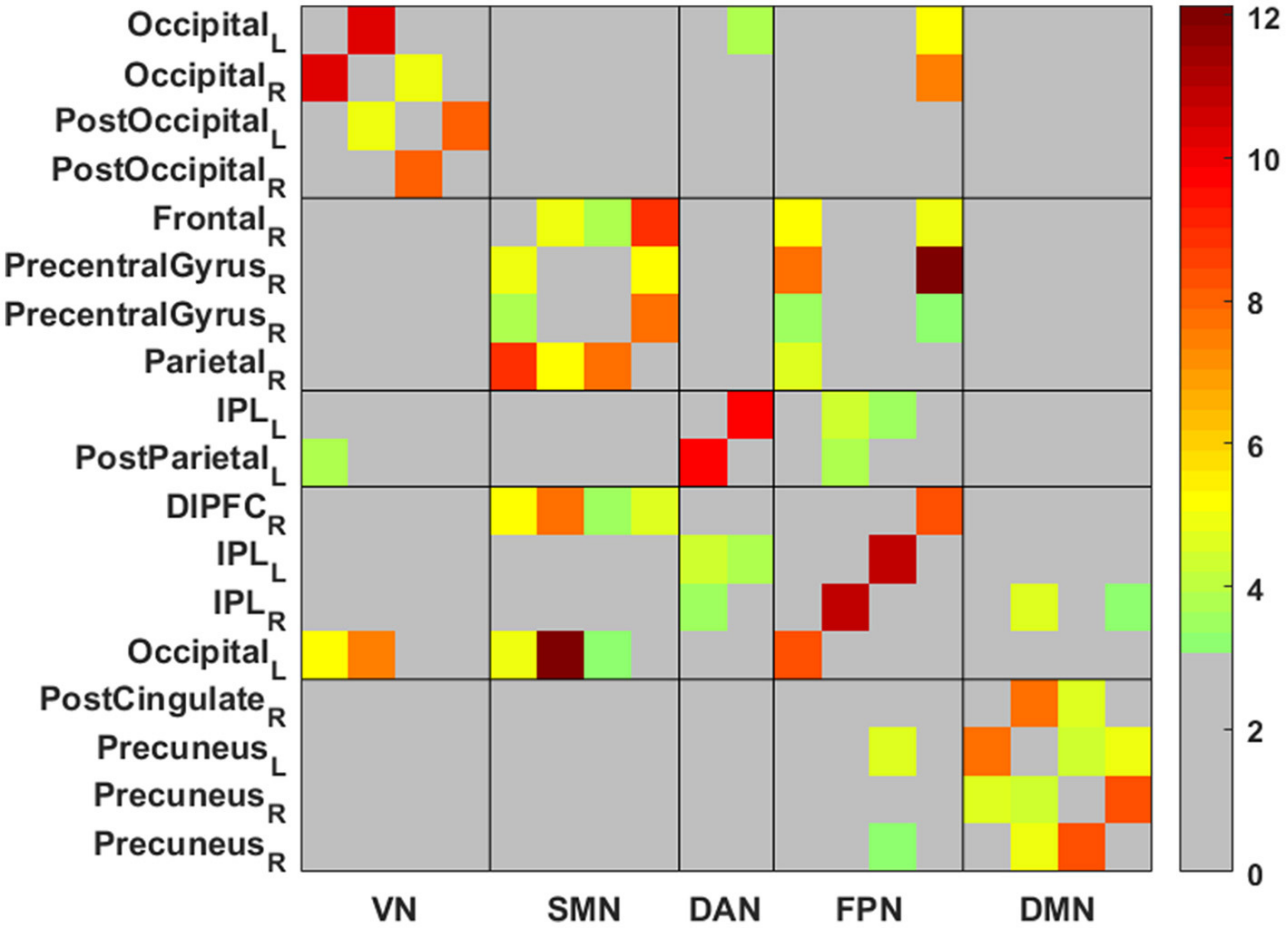
The matrix of network functional connectivity (FC) differences between post- vs. pre-intervention. Color bar represents Fisher's *z*-transformed Pearson correlation coefficient. VN, visual network; SMN, somatosensory network; DAN, dorsal attention network; DMN, default mode network; FPN, frontoparietal network; L, left; R, right. Shown are 18 × 18 regions matrix (all are within the five networks) with color shading indicating statistically significant results. The network FC for the pair of regions with significant results were calculated as a Pearson's linear correlation coefficient, which was then Fisher-z transformed to derive a symmetric z score matrix that represented the functional connectivity network.

**Figure 2:**
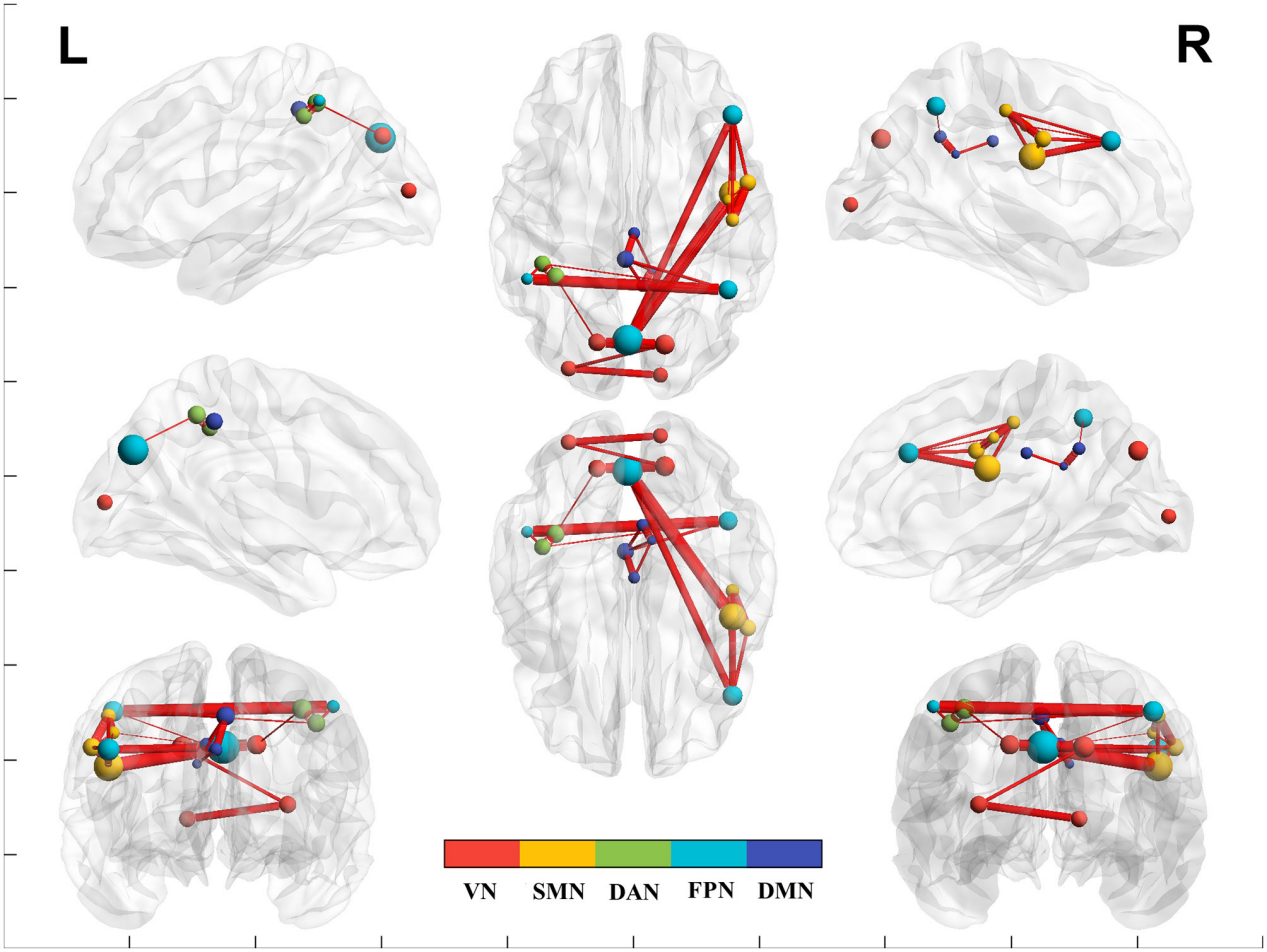
The brain network functional connectivity (FC) between post- vs. pre-intervention. Red line indicates increased network FC post- than pre-intervention. VN, visual network; SMN, somatosensory network; DAN, dorsal attention network; DMN, default mode network; FPN, frontoparietal network.

Table 2 and Figures 1, 2 present group average results and demonstrate significant intra-network FC increases in multiple networks following TLNS intervention. Specifically, within the somatosensory network (SMN), significantly increased FC was observed between the right frontal gyrus and right parietal lobule (*t* = 8.904, *p* = 0.002), as well as between the right precentral gyrus and right parietal lobule (*t* = 7.817, *p* = 0.002). The default mode network (DMN) also showed significantly increased connectivity between the right posterior cingulate cortex and left precuneus (*t* = 7.762, *p* = 0.002). Additionally, intra-network FC within the frontoparietal network (FPN) significantly increased between the left and right inferior parietal regions (*t* = 10.767, *p* = 0.001) and between the right dorsal prefrontal cortex and left occipital gyrus (*t* = 8.261, *p* = 0.002). The visual network (VN) also exhibited significantly increased connectivity, particularly between bilateral regions of the occipital gyrus (*t* = 10.247, *p* = 0.002).

**Table 2:** The significant network functional connectivity differences between post- vs. pre-intervention.

**Regions**		**Networks**		***t*-value**	***p*-value**
R frontal	R parietal	SMN	SMN	8.904	0.002
R precentral	R parietal	SMN	SMN	7.817	0.002
R post cingulate	L precuneus	DMN	DMN	7.762	0.002
R precuneus	R precuneus	DMN	DMN	8.323	0.002
L inferior parietal	R inferior parietal	FPN	FPN	10.767	0.001
R dorsal prefrontal	L occipital	FPN	FPN	8.261	0.002
L occipital	R occipital	VN	VN	10.247	0.002
L post occipital	R post occipital	VN	VN	7.892	0.002
L inferior parietal	L post parietal	DAN	DAN	9.819	0.002
R precentral gyrus	L occipital	SMN	FPN	12.136	0.001
R precentral gyrus	R dorsal prefrontal	SMN	FPN	7.768	0.002

Positive *t*-value means post- is greater than pre-interventions. Paired *t*-test FDR *p* < 0.05.

VN, visual network; SMN, somatosensory network; DAN, dorsal attention network; DMN, default mode network; FPN, frontoparietal network; L, left; R, right.

### 3.3 Inter-network functional connectivity

Table 2 and Figures 1, 2 also present group average results and demonstrate significant inter-network FC increases from pre-intervention to post-intervention in several key networks. Notably, the inter-network FC between the SMN and FPN was significantly increased, particularly between the right precentral gyrus and left occipital gyrus (*t* = 12.136, *p* = 0.001), and between the right precentral gyrus and right dorsal prefrontal cortex (*t* = 7.768, *p* = 0.002).

In addition to the above five networks, there were no significant inter- and intra-network differences between VAN, SC, and cerebellum networks before and after the intervention.

### 3.4 Correlation

Correlation analyses demonstrated a significant negative correlation between changes in SOT scores and inter-network FC between the right dorsolateral prefrontal cortex (FPN) and right precentral gyrus (SMN) (*r* = −0.784, *p* = 0.012), suggesting that greater reductions in FC between these networks were associated with better balance performance. In contrast, a significant positive correlation was found between changes in DGI scores and intra-network FC within the SMN, specifically between the right precentral and right parietal lobule (*r* = 0.728, *p* = 0.026), suggesting that increases in connectivity within the somatosensory network were linked to improvements in gait performance. Details are shown in Table 3.

**Table 3:** The correlations between edge-based FC (post minus pre) and behavioral scores (post minus pre).

**Regions**	**Networks**	**Behavioral task**	***r* (9)**	***p***
R dorsolateral prefrontal and R precentral	FPN and SMN	SOT	−0.784	0.012
R precentral and R parietal	SMN and SMN	DGI	0.728	0.026

FPN, frontoparietal network; SMN, somatosensory network; L, left; R, right.

## 4 Discussion

After TLNS intervention on mmTBI patients, our study revealed significant increased intra-network FC at the VN, DMN, DAN, FPN, and SMN, and inter-network FC between the SMN and FPN. Additionally, mmTBI patients demonstrated significantly increased performance on the SOT and DGI tests after TLNS intervention compared to pre-TLNS intervention. There were significant positive correlations between the DGI and intra-network FC within the SMN, and significant negative correlations of the SOT and inter-network FC between the FPN and the SMN.

The SMN, which includes regions such as the precentral gyrus, dorsolateral and dorsomedial prefrontal cortex, temporoparietal junction (Kim et al., 2019; Kucyi et al., 2012), are responsible for functions such as motor, auditory, and somatosensory processing (Kim et al., 2019; Li et al., 2021). Post-TLNS intervention in our study showcased increased intra- and internetwork FC at the SMN, indicating that TLNS therapy may be effective in increasing motor perceptions, auditory functions, somatosensory processing, and vestibular-visual interactions that are crucial for regulating gait and balance. The VN encompasses the primary and second visual cortex that refers to visual attention (Macaluso et al., 2000), motor perception (Di Plinio and Ebisch, 2018; Li et al., 2021) and control monitoring (Brandi, 2014; Brandi et al., 2014). Post-TLNS intervention mmTBI exhibited significantly increased scores on SOT and DGI performances and increased VN FC, demonstrating improved abilities in sensory perception in motor and vision.

The post-TLNS intervention results also exhibited increased intra-network FC within the DAN, increased intra-network FC within the FPN, and increased inter-network FC between FPN and the SMN. The DAN is closely adjacent to the SMN and VN (Eklund et al., 2016; Li et al., 2021; Yan et al., 2019; Yeo et al., 2011) and is responsible for visuo-spatial processing (Li B. et al., 2013; Yan et al., 2019), containing neurons with spatially organized receptive fields (Posner et al., 2013; Wise et al., 2017) that are important to attentional shifting (Davey et al., 2012; Desseilles et al., 2009), goal-directed tasks (Fox et al., 2005) and visual-guided actions (Iwabuchi et al., 2015). Moreover, the DAN also plays a role in perceptual attention that is modulated by the FPN (Yan et al., 2019). The FPN includes regions such as dorsolateral prefrontal, parietal and occipital regions (Di Plinio and Ebisch, 2018) and it is important for executive control that refers to processes such as monitoring or flexible shifting in order to facilitate visual and movement interactions (Yan et al., 2019). Inter-network increased FC between FPN and SMN shown in our study after TLNS intervention may be especially effective in improving the cognitive functions that the FPN and SMN are responsible for.

The DMN has been widely studied with its alteration in TBI patients (D'Souza et al., 2020; Johnson et al., 2012). The DMN includes the regions such as the posterior cingulate, medial prefrontal and precuneus cortex (Washington and VanMeter, 2015), and it supports functions such as cognition (Cabeza et al., 2004; Hampson et al., 2006), attention regulation (Hampson et al., 2006; Leech et al., 2011), introspection, memory encoding, and error recognition (Mason et al., 2007; Zhou et al., 2012). The FC between dorsolateral prefrontal cortex and precuneus refers to the mental imagery, episodic memory and visuospatial imagery (Cavanna and Trimble, 2006; Margulies et al., 2009). The increased DMN FC after TLNS intervention in our study may reflect the improved ability of cognitive control or attentional regulation in mmTBI patients.

The intra-network FC changes (post- minus pre-intervention) in SMN was positively correlated to the changes of behavioral DGI testing (also the post- minus pre-intervention), which illustrates that more FC changes in SMN after intervention were associated with higher behavior improvements that are related to gait and stability. However, the inter-network FC changes between the FPN and SMN were negatively correlated to the changes of behavioral SOT testing, suggesting that fewer internetwork FC changes were associated with better performance. This possibly reflects the greater efficiency or automatization of functions such as somatosensory, vision, or vestibular balance involved after the TLNS intervention and less executive and attentional control by FPN. Moreover, the other potential network FCs or brain regions not included in Table 1 may have a compensatory role, or reflect the adaptive plasticity of functional connectivity as a result of TLNS intervention (Guvenc et al., 2018).

It is worth noting that in our previous study investigating the role of specific ROIs, no significant correlations were observed between the sensory/somatomotor, visual and cerebellar regions and the SOT and DGI (Hou et al., 2022). However, the current study showed significant correlations between SOT or DGI and network FC.

The limitations of the present study include a small sample size. To overcome this limitation, future studies should include larger sample sizes in order to improve generalizability and increase statistical power, as well as to discern gender differences between males and females. Additionally, participants in the main study received either a low- or high-frequency pulse (LFP or HFP) tongue stimulation, so a follow-up study of the specific effects of LFP or HFP on individuals will be beneficial. However, our study, while adding to our previous work (Hou et al., 2022) demonstrates that a network-based approach to study whole brain interactions yields promising results in terms of identifying significant relationships between functional connectivity changes and outcome measures.

In summary, the present study showed evidence that changes to brain network functional connectivity involved in gait, balance and motor control were produced by TLNS which improves functions of balance and gait in patients that have chronic symptoms from mild-moderate traumatic brain injury.

## 5 Conclusion

This study demonstrates that translingual neural stimulation (TLNS), in combination with physical therapy, leads to significant improvements in balance and gait in patients with mmTBI. These behavioral improvements, as measured by the SOT and DGI, are accompanied by increased intra- and inter-network RSFC in key brain networks, including the visual, default mode, dorsal attention, frontoparietal and somatosensory networks. The results suggest that TLNS may enhance brain network plasticity, offering a promising therapeutic approach for addressing mmTBI-related functional deficits. Future studies with larger sample sizes and longer follow-up periods are needed to validate these findings and further explore the mechanisms underlying TLNS-induced neuroplasticity.

## Data Availability

The raw data supporting the conclusions of this article will be made available by the authors, without undue reservation.
